# Sacral Dysmorphism as an Independent Risk Factor for Cortical Breach During Percutaneous S1 Iliosacral Screw Fixation

**DOI:** 10.3390/jcm15103847

**Published:** 2026-05-16

**Authors:** Cemil Aktan, Muhammed Ergün, Melih Ünal, Nevfel Kahvecioğlu, Cemal Hasoğlan, Yusuf Alper Katı, Halil Yalçın Yüksel

**Affiliations:** 1 Antalya Eğitim ve Araştırma Hastanesi, Antalya 07600, Türkiye; ergunmuhammed_07@hotmail.com (M.E.); meliih.unal@gmail.com (M.Ü.); nevfelkahvecioglu@hotmail.com (N.K.); 2 Department of Orthopedics and Traumatology, Gazimağusa State Hospital, Gazimagusa 99450, Cyprus; 3 Department of Orthopedics and Traumatology, Private Anatolia Hospital, Antalya 07400, Türkiye; alperkati@gmail.com (Y.A.K.);

**Keywords:** sacral dysmorphism, iliosacral screw, cortical breach, pelvic fracture, sacroiliac fixation, S1 corridor, computed tomography

## Abstract

**Background/Objectives**: Sacral dysmorphism is a common anatomical variant that may significantly affect the safety of percutaneous iliosacral screw fixation. Although its morphological characteristics are well described, its impact on clinically relevant outcomes—particularly cortical breach during S1 screw placement—remains insufficiently defined. This study aimed to evaluate whether sacral dysmorphism is an independent risk factor for cortical breach during percutaneous S1 iliosacral screw fixation. **Methods**: This retrospective cohort study included 112 adult patients with sacral fractures treated with percutaneous S1 iliosacral screw fixation between January 2018 and December 2024. Sacral dysmorphism was defined on preoperative CT scans using qualitative features, with ≥2 criteria required for classification as dysmorphic. Quantitative morphometric parameters, including S1 osseous corridor width and screw anteversion angle, were also measured. The primary outcome was the presence of cortical breach on postoperative CT imaging. Multivariate logistic regression analysis was performed to evaluate the independent association between sacral dysmorphism and cortical breach. **Results**: Sacral dysmorphism was identified in 32 patients (28.6%). Cortical breach occurred in 19 patients (17.0%) and was significantly more frequent in the dysmorphic group compared with the non-dysmorphic group (34.4% vs. 10.0%; *p* = 0.002). Sacral dysmorphism was independently associated with cortical breach (adjusted OR: 4.38; 95% CI: 1.42–13.50; *p* = 0.010). Dysmorphic sacra demonstrated significantly narrower S1 osseous corridors (16 mm vs. 25 mm; *p* < 0.001) and greater screw anteversion angles (28° vs. 9°; *p* < 0.001). Breach severity was also significantly greater in dysmorphic patients (*p* = 0.003). **Conclusions**: Sacral dysmorphism is an independent risk factor for cortical breach during percutaneous S1 iliosacral screw fixation. The geometric constraints of dysmorphic sacra, including corridor narrowing and increased anteversion requirements, significantly compromise screw placement safety. Careful CT-based evaluation and individualized trajectory planning are essential to optimize fixation outcomes in this high-risk anatomical subgroup.

## 1. Introduction

The sacrum plays a key biomechanical role in transmitting axial loads from the spine to the lower extremities through the sacroiliac (SI) joint [1]. Sacral fractures account for approximately 30–45% of pelvic ring injuries and most frequently involve the S1–S2 region, where load transfer occurs in close proximity to critical neural and vascular structures [2,3,4]. Consequently, percutaneous iliosacral screw fixation has become a widely adopted technique for posterior pelvic stabilization. However, the procedure remains technically demanding because safe screw placement requires accurate interpretation of sacral morphology, precise fluoroscopic visualization, and strict control of screw trajectory.

Sacral dysmorphism is a common anatomical variant, reported in up to one-third of individuals, and represents a key factor influencing the safety of iliosacral screw placement. Dysmorphic sacra exhibit characteristic morphological features, including altered osseous corridors, mammillary processes, a tongue-in-groove sacroiliac joint configuration, persistence of the S1–S2 disc space, and irregular neural foramina [5,6,7]. The reported prevalence of sacral dysmorphism varies across populations and imaging-based definitions, reflecting both anatomical heterogeneity and differences in diagnostic criteria. These anatomical variations may substantially narrow or even eliminate the safe S1 osseous corridor, thereby increasing the risk of screw malposition, cortical breach, and iatrogenic neurovascular injury.

From a surgical perspective, the clinical relevance of sacral dysmorphism lies in its direct effect on screw trajectory planning. In dysmorphic sacra, the S1 osseous corridor may be narrower, more oblique, or less predictable, requiring greater screw anteversion to maintain an intraosseous path. These geometric constraints may increase the risk of anterior cortical violation, which is particularly important because of the proximity of major vascular structures and lumbosacral nerve roots. Therefore, detailed preoperative CT-based assessment of sacral morphology is essential for identifying high-risk anatomy and selecting an individualized fixation strategy.

Previous studies have primarily focused on describing the anatomical constraints of dysmorphic sacra and their implications for surgical feasibility, with some authors advocating alternative fixation strategies such as S2 screw placement in selected cases [8]. However, despite growing recognition of sacral dysmorphism as a technical challenge, there remains limited clinical evidence directly linking dysmorphic morphology to measurable postoperative outcomes, particularly cortical breach during S1 iliosacral screw fixation.

A more precise understanding of how sacral dysmorphism affects screw trajectory and breach risk is essential for preoperative planning and intraoperative decision-making. Identifying dysmorphism as an independent predictor of cortical violation may enable improved risk stratification and more individualized fixation strategies in patients with posterior pelvic ring injuries.

Therefore, the aim of this study was to evaluate the association between sacral dysmorphism and cortical breach during percutaneous S1 iliosacral screw fixation using CT-based morphologic assessment. We hypothesized that sacral dysmorphism would be independently associated with an increased risk of cortical breach, reflecting the geometric limitations of the S1 osseous corridor in dysmorphic sacra.

## 2. Materials and Methods

### 2.1. Study Design and Patient Selection

This retrospective cohort study included adult patients admitted to our Level I trauma center with sacral fractures between January 2018 and December 2024. Demographic characteristics, clinical data, and imaging studies were retrieved from the institutional digital archive system.

Inclusion criteria were: (1) age ≥ 18 years, (2) availability of preoperative and postoperative pelvic computed tomography (CT) scans with sufficient resolution for detailed morphologic assessment, and (3) a minimum postoperative follow-up of 12 months.

**Figure 1:**
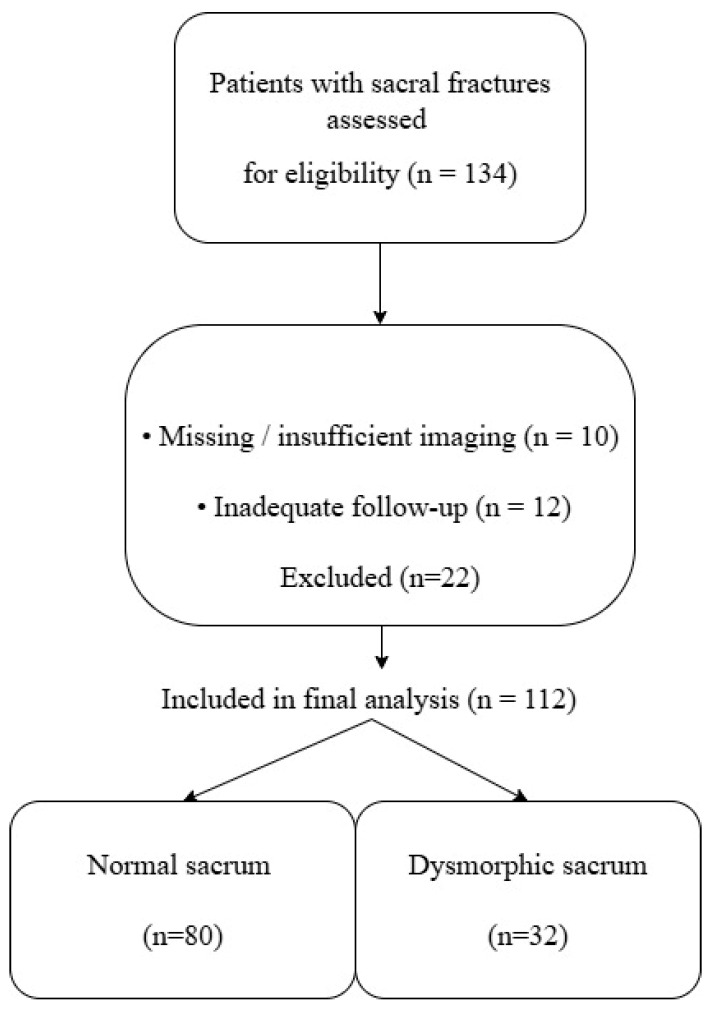
Flowchart of patient selection and group allocation showing inclusion, exclusion, and distribution of patients into normal and dysmorphic sacrum groups.

Patients were excluded if they had inadequate or missing CT imaging, insufficient follow-up, pathological fractures, prior sacral instrumentation, or incomplete medical records (Figure 1).

To ensure procedural consistency, only patients treated with percutaneous S1 iliosacral screw fixation in the supine position were included in the analysis.

Ethics approval was obtained from the Institutional Review Board (Decision No: 12/3; Date: 17 July 2025). The requirement for informed consent was waived due to the retrospective design. All patient data were anonymized, and the study was conducted in accordance with the Declaration of Helsinki.

Only patients with complete clinical and radiological data were included in the final analysis.

### 2.2. Radiologic Evaluation

**Figure 2:**
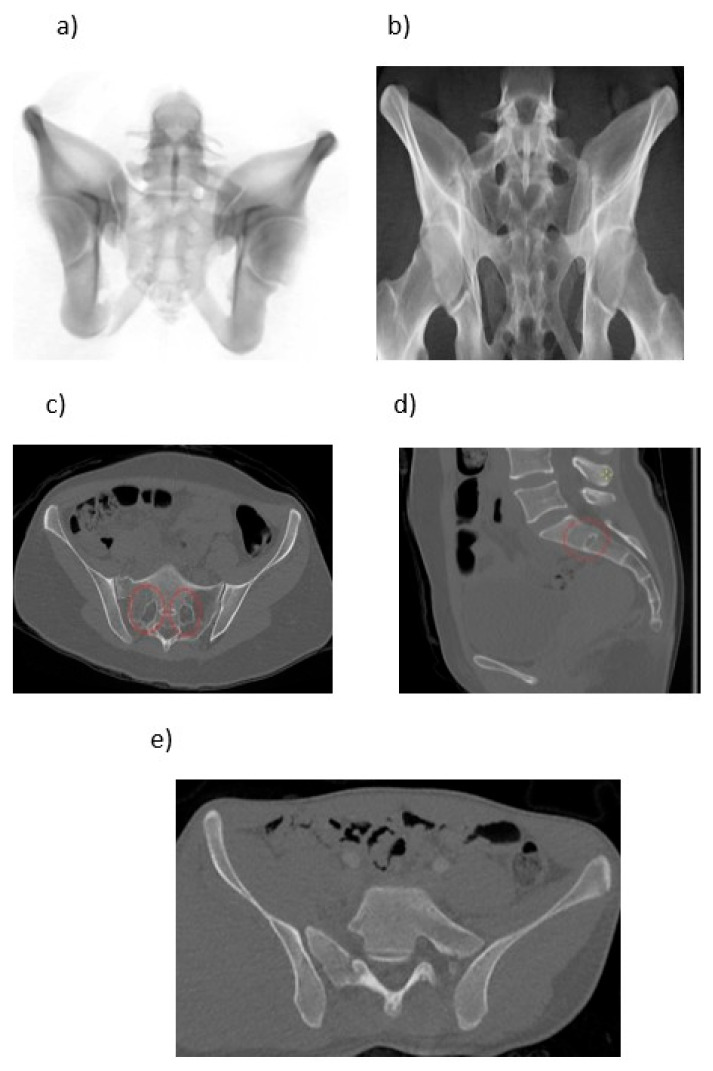
Qualitative radiographic features of sacral dysmorphism on pelvic outlet and CT images. (**a**) Crest–disc colinearity, (**b**) oblique alar transverse process, (**c**) noncircular S1 foramina, (**d**) S1–S2 residual disc, and (**e**) steep alar slope.

Preoperative pelvic CT scans were used to assess sacral morphology. Sacral dysmorphism was defined based on qualitative CT-based morphologic features described by Routt et al. [9] The evaluated criteria included: (1) colinearity, (2) oblique residual transverse processes, (3) noncircular S1 neural foramina, (4) residual S1–S2 disc space, and (5) steep sacral alar slopes [9] (Figure 2).

Given that sacral dysmorphism represents a morphological spectrum rather than a binary entity, a pragmatic threshold was applied to improve reproducibility. Sacra demonstrating two or more of these features were classified as dysmorphic, whereas those with fewer than two features were considered non-dysmorphic.

Two orthopedic trauma surgeons independently reviewed all scans using 1 mm slice-thickness CT images with multiplanar reconstruction (MPR) on the institutional PACS system. Both reviewers had more than 5 years of experience in pelvic trauma surgery, including the evaluation, treatment planning, and surgical management of pelvic injuries, and were familiar with CT-based sacral morphology assessment. Interobserver reliability was assessed using Cohen’s kappa coefficient and interpreted according to the Landis and Koch classification. In cases of disagreement, a consensus reading was performed to reach a final classification.

To provide an objective morphometric characterization, quantitative parameters described by Kaiser et al. [10] were additionally measured, including measurements S1 osseous corridor width, sacral alar slope, anterior cortical encroachment, upper sacral tilt, and interforaminal dimensions. These measurements were used to further characterize corridor geometry relevant to screw trajectory planning and were not applied as standalone diagnostic criteria for dysmorphism. All quantitative assessments were performed by a fellowship-trained musculoskeletal radiologist blinded to group allocation and clinical outcomes.

**Figure 3:**
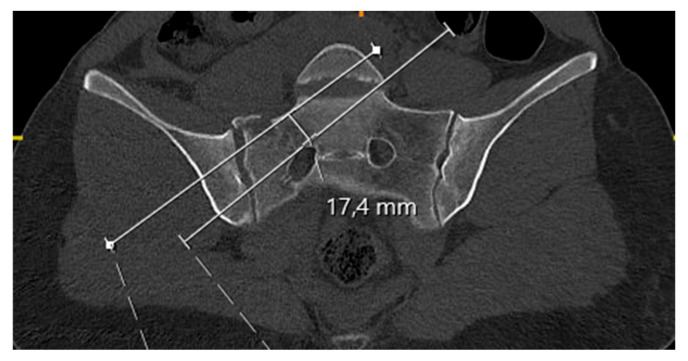
Preoperative measurement of the narrowest width of the S1 osseous corridor on coronal CT.

The narrowest width of the S1 osseous corridor was measured on preoperative true-coronal MPR images orthogonal to the sacral axis, following established techniques [11,12] (Figure 3). The minimum corridor diameter across the S1 segment was recorded for analysis.

**Figure 4:**
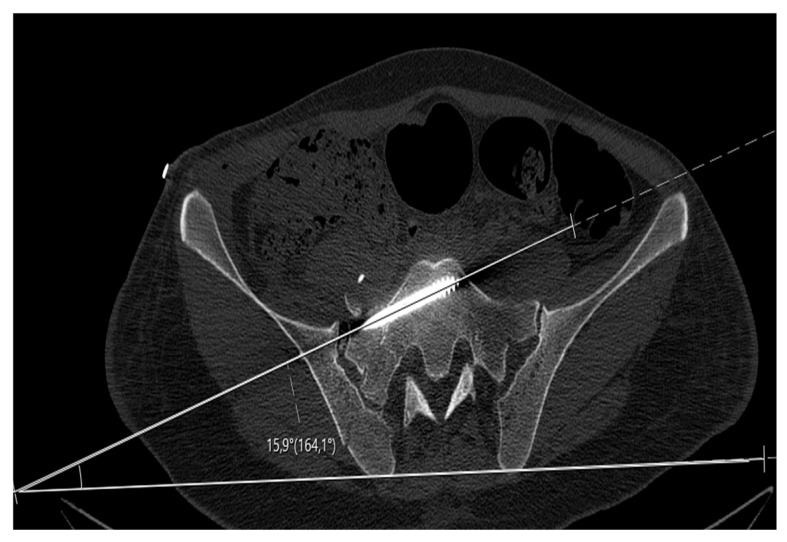
Postoperative measurement of the S1 screw anteversion angle on axial CT.

Postoperative screw anteversion was measured on axial CT reformats. The anteversion angle was defined as the angle between the central screw axis and a reference line connecting the bilateral posterior superior iliac spines (PSIS) (Figure 4). Measurements were obtained on true-axial images aligned parallel to the superior sacral endplate to ensure standardized orientation. Anteversion angle was included as a quantitative parameter reflecting screw trajectory orientation, given its established association with anterior cortical breach and potential neurovascular risk.

### 2.3. Surgical Technique

All procedures were performed with the patient in the supine position under general anesthesia on a radiolucent operating table. Closed reduction was achieved using longitudinal traction and gentle internal or external rotation as required. A mobile single-plane fluoroscopy unit was used to obtain standardized anteroposterior, inlet, outlet, and true lateral sacral views. Adequate inlet and outlet projections were confirmed by symmetric visualization of the sacral ala and neural foramina.

On the true lateral sacral view, the iliocortical density (ICD) line served as the primary landmark for identifying the entry zone for S1 iliosacral screw placement. The entry point was selected at the midpoint of the ICD contour, aligned with the planned intraosseous trajectory targeting the S1 vertebral body.

After lateral localization, a percutaneous guidewire (K-wire) was advanced under fluoroscopic guidance. Wire position was sequentially verified on inlet and outlet views to avoid anterior encroachment or cortical violation and to ensure an intraosseous trajectory within the S1 corridor. Screw length was determined using a second guidewire of identical length placed alongside the first. When necessary, cannulated drilling was performed over the guidewire, and a 7.3 mm partially threaded cannulated screw was inserted under continuous fluoroscopic control.

All procedures were performed by a dedicated trauma team and supervised by senior orthopedic trauma surgeons with substantial experience in iliosacral screw fixation, following a standardized operative protocol to ensure procedural consistency and minimize technique-related variability.

### 2.4. Postoperative Assessment

Postoperative data were obtained retrospectively from electronic medical records and the institutional imaging archive. As part of routine clinical practice at our institution, pelvic CT scans were obtained on the first postoperative day following iliosacral screw fixation and were used for postoperative radiologic analysis.

The primary outcome of the study was the presence of cortical breach on postoperative CT imaging. Secondary radiologic parameters included breach direction, breach depth, and screw anteversion angle.

All postoperative CT images were reviewed using multiplanar reconstruction (MPR) in axial, sagittal, and coronal planes. Cortical breach was classified according to the direction of cortical violation (anterior, posterior, superior, inferior, or lateral cortical exit). In addition, screws demonstrating an extraosseous segment with subsequent re-entry into the osseous corridor were categorized separately as an “in–out–in” trajectory pattern rather than a directional cortical breach. The extent of cortical violation was quantified in millimeters as the maximal perpendicular extrusion beyond the cortical boundary. Breach severity was further categorized by depth, with violations exceeding 2 mm considered clinically relevant, in accordance with previously described CT-based grading systems [13].

All postoperative CT evaluations were performed by a fellowship-trained musculoskeletal radiologist blinded to group allocation and intraoperative details. Early postoperative complications were identified through chart review and included new neurological deficits, screw malposition requiring clinical attention, implant migration or loosening, and unplanned revision surgery.

Radiological outcomes were compared between patients with dysmorphic and non-dysmorphic sacra to evaluate the impact of sacral morphology on cortical breach risk following S1 iliosacral screw fixation.

As part of the institutional protocol, all patients underwent routine postoperative pelvic CT imaging on the first postoperative day, regardless of clinical symptoms. This standardized imaging approach enabled systematic detection of cortical breach across the entire cohort.

In cases where cortical breach was identified, postoperative management was guided by a symptom and risk-based approach incorporating both clinical findings and breach direction. Patients were closely monitored for neurological deficits, radicular symptoms, or other procedure-related complications. In the absence of clinical symptoms, a conservative management strategy with close observation was adopted. In contrast, the presence of neurological findings or high-risk breach patterns, particularly anterior cortical violation, would prompt further evaluation and consideration of revision surgery. In the present cohort, no patients met the criteria for surgical revision, and all cases were managed conservatively.

### 2.5. Statistical Analysis

All analyses were carried out using SPS software (Version 26.0; IBM Corp., Armonk, NY, USA). The distribution of continuous variables was examined using both graphical methods (histograms) and the Shapiro–Wilk test. Data with a normal distribution are reported as mean ± standard deviation, whereas skewed data are presented as median with interquartile range (IQR). Categorical variables are summarized as counts and percentages.

Differences between patients with dysmorphic and non-dysmorphic sacral morphology were evaluated using statistical tests appropriate to the data distribution. The independent samples *t*-test was applied for normally distributed variables, whereas the Mann–Whitney U test was used for non-normally distributed variables. For categorical variables, either the chi-square test or Fisher’s exact test was performed as appropriate. Interobserver agreement for the classification of sacral dysmorphism was assessed using Cohen’s kappa coefficient and interpreted according to the Landis and Koch scale.

The association between sacral dysmorphism and cortical breach was examined using binary logistic regression analysis. Cortical breach was defined as the dependent variable, and sacral morphology was included as the primary predictor. Additional variables, including age and sex as well as those showing baseline differences, were incorporated into the model to adjust for potential confounding. Multicollinearity among predictors was evaluated prior to model construction.

Model performance was assessed using the Hosmer–Lemeshow goodness-of-fit test. The results are presented as odds ratios (ORs) with 95% confidence intervals (CIs).

All statistical tests were two-sided, and a *p*-value < 0.05 was considered statistically significant.

## 3. Results

A total of 112 patients were included in the study. The dysmorphic group was significantly older than the non-dysmorphic group (51.5 ± 17.8 vs. 36.6 ± 15.4 years; *p* < 0.001), whereas sex distribution was comparable between groups (female proportion: 43.8% vs. 31.2%; *p* = 0.301) (Table 1).

Interobserver assessment demonstrated almost perfect agreement for the identification of sacral dysmorphism (κ = 0.825; 95% CI: 0.694–0.934; 92.9% agreement). Overall, 32 patients (28.6%) were classified as having a dysmorphic sacrum.

Cortical breach occurred in 19 patients (17.0%). The prevalence of breach was significantly higher in the dysmorphic group compared with the non-dysmorphic group (34.4% vs. 10.0%; *p* = 0.002), corresponding to an unadjusted odds ratio (OR) of 4.76 (95% CI: 1.78–12.72). After adjustment for age, sacral dysmorphism remained independently associated with cortical breach (adjusted OR: 4.38; 95% CI: 1.42–13.50; *p* = 0.010). No cases of new postoperative neurological deficits, screw malposition requiring intervention, or unplanned reoperation were observed, including among patients with cortical breach.

Among the 19 patients with cortical breach, 11 had dysmorphic sacra and 8 had normal sacral morphology. In the dysmorphic group, anterior cortical breach was the most frequent directional breach (8/11, 72.7%), whereas 3 patients demonstrated an in–out–in trajectory pattern. In contrast, among patients with normal sacra, lateral cortical exit predominated (7/8, 87.5%), with only one case demonstrating an in–out–in trajectory. No posterior or superior cortical breaches were observed in either group.

Despite the presence of cortical breach, including cases with greater breach depth (≥3 mm), no patients demonstrated new postoperative neurological deficits or required revision surgery during the hospitalization period or throughout the minimum 12-month follow-up period.

**Figure 5:**
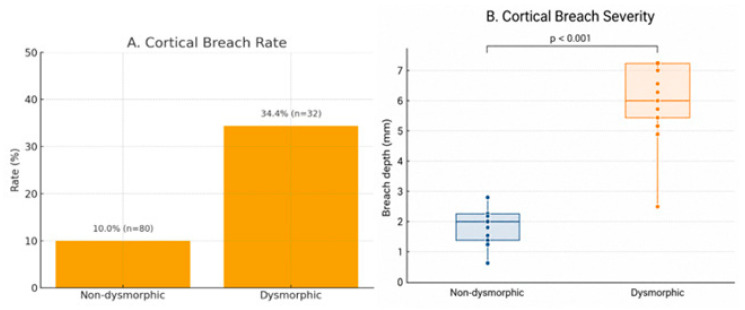
(**A**) The prevalence of cortical breach was significantly higher in the dysmorphic group (*p* = 0.002). (**B**) Breach severity was significantly greater in dysmorphic sacra (*p* < 0.001). Data are presented as percentage rates (**A**) and as box-and-whisker plots showing medians and interquartile ranges with individual data points overlaid (**B**). Only patients with cortical breach were included in the severity analysis.

Both the prevalence and severity of cortical breach were significantly higher in dysmorphic sacra (*p* = 0.002 and *p* < 0.001, respectively), with substantially greater breach depths observed in dysmorphic patients (Figure 5A,B).

**Figure 6:**
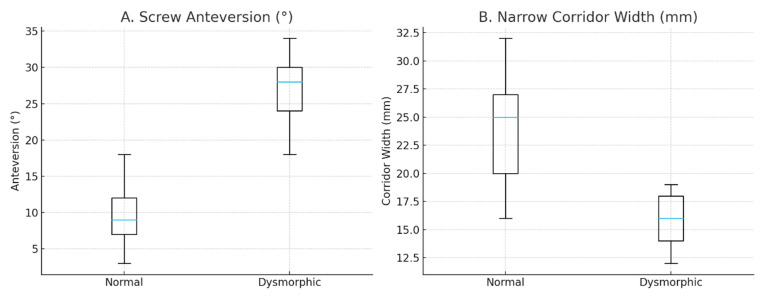
Effect of sacral morphology on screw anteversion and osseous corridor width. (**A**) Screw anteversion was significantly higher in dysmorphic sacra (*p* < 0.001). (**B**) The narrowest corridor was significantly reduced in dysmorphic sacra (*p* < 0.001). Data are presented as medians and interquartile ranges. Figure 6 presents pooled data for each group irrespective of sex, whereas Table 1 provides sex-stratified subgroup analyses.

Patients with a dysmorphic sacrum demonstrated significantly greater screw anteversion compared with those with normal morphology (median [IQR]: 28° (24–30) vs. 9° [7,8,9,10,11,12]; *p* < 0.001). In addition, the narrowest S1 osseous corridor width was significantly reduced in the dysmorphic group (median [IQR]: 16 mm [14,15,16,17,18] vs. 25 mm (20–27); *p* < 0.001), indicating a substantial anatomical constraint of the S1 corridor in dysmorphic sacra (Figure 6).

Sex-based subgroup analyses suggested narrower corridor dimensions in female patients (Table 1), although these findings should be interpreted with caution given the limited sample size.

## 4. Discussion

The principal finding of the present study is that sacral dysmorphism is independently associated with an increased risk of cortical breach during percutaneous S1 iliosacral screw fixation. Patients with dysmorphic sacral morphology demonstrated both a significantly higher incidence and greater severity of cortical violation compared with those with normal sacra, and this relationship persisted after adjustment for age. These findings indicate that sacral dysmorphism is not merely an anatomical variation but a clinically relevant risk factor affecting screw placement safety. The observed increase in breach risk is likely attributable to the geometric constraints of dysmorphic sacra, including a substantially narrowed S1 osseous corridor and the requirement for greater screw anteversion to maintain an intraosseous trajectory.

In addition to the overall incidence of cortical breach, the pattern and direction of violation provide important clinical insight. In the present study, anterior cortical violations were predominantly observed in dysmorphic sacra, likely reflecting the increased anteversion required to maintain an intraosseous trajectory in a narrowed S1 corridor. This finding is clinically important, as anterior breach carries a higher risk of injury to major vascular structures and lumbosacral nerve roots.

In contrast, breaches observed in normal sacra were more frequently lateral and involved exit through the contralateral sacral cortex, which may represent a relatively lower-risk pattern in terms of critical neurovascular structures. The higher prevalence of anterior breach in dysmorphic sacra further supports the concept that altered sacral geometry directly influences not only the risk but also the pattern of screw malposition.

From a clinical perspective, these findings have direct implications for intraoperative decision-making. In patients with dysmorphic sacra, the increased likelihood of anterior cortical breach underscores the importance of cautious trajectory planning, particularly with respect to screw anteversion. Surgeons should maintain a lower threshold for intraoperative trajectory adjustment or alternative fixation strategies in cases where anterior encroachment is suspected. This direction-specific risk profile further supports the role of detailed preoperative CT-based planning in minimizing neurovascular complications.

Although no clinically significant postoperative symptoms were observed in patients with cortical breach, these findings should be interpreted with caution. The absence of immediate clinical manifestations does not necessarily indicate the absence of risk, and cortical breach—particularly in high-risk directions—should not be considered benign. Careful clinical monitoring remains essential, particularly in cases involving high-risk breach directions.

Based on the findings of the present study and our institutional experience, we propose a practical postoperative management approach. From a practical standpoint, postoperative management can be conceptualized as a stepwise process beginning with radiographic detection, followed by clinical assessment, and subsequently stratified according to symptom status and breach characteristics. In patients with radiographically detected cortical breach, clinical assessment should guide management decisions. Asymptomatic patients can be managed conservatively with close observation and serial clinical follow-up. In contrast, the presence of neurological symptoms, radicular pain, or high-risk breach patterns, particularly anterior cortical violation, should prompt further evaluation and consideration of revision surgery. This symptom- and pattern-based approach may help optimize patient safety while avoiding unnecessary interventions.

Sacral dysmorphism is a common anatomical variant with clinically relevant implications for iliosacral screw fixation. In the present cohort, dysmorphic morphology was identified in 28.6% of patients, consistent with previously reported prevalence rates of up to 40% [5,6,7]. Dysmorphic sacra are characterized by alterations in upper sacral morphology that may substantially narrow the S1 osseous corridor, thereby increasing the technical complexity of achieving a safe intraosseous trajectory. In line with these anatomical constraints, our findings demonstrated significantly greater anteversion requirements and reduced corridor dimensions in dysmorphic sacra, highlighting the geometric limitations that must be addressed during screw placement. Previous studies have emphasized these challenges and proposed alternative fixation strategies, including S2 screw placement, in selected cases [8]. Taken together, these observations reinforce the importance of recognizing sacral dysmorphism during preoperative CT evaluation and integrating these anatomical considerations into trajectory planning to minimize the risk of cortical breach.

Although S2 fixation has frequently been proposed as an alternative strategy in dysmorphic sacra, its safety profile remains a matter of debate. Several studies have reported higher malposition rates and more limited fluoroscopic visualization for S2 screws compared with S1 fixation [14,15,16], and Van den Bosch et al. described an increased incidence of neurological complications associated with S2 screw placement [17]. In this context, the increased breach risk observed in dysmorphic sacra in the present study suggests that no fixation segment can be considered universally safe, and that anatomical constraints must be carefully evaluated for each patient. Accordingly, segment selection should remain individualized, guided by patient-specific sacral morphology and supported by meticulous preoperative planning and intraoperative imaging.

Iliosacral screw fixation remains a technically demanding procedure that requires precise trajectory planning to ensure safe intraosseous placement [18,19,20]. In line with previous CT-based morphometric studies, dysmorphic sacra have been consistently associated with a narrowed S1 osseous corridor and increased anteversion requirements [21]. Our findings extend these observations by demonstrating that these geometric constraints translate into clinically meaningful increases in cortical breach risk. Together with prior reports indicating that a safe S1 corridor may be absent or markedly restricted in a substantial proportion of dysmorphic sacra [6,8,22,23], these results support the concept that altered sacral geometry represents a critical determinant of fixation safety. Although greater screw anteversion and narrower S1 corridor width were significantly associated with dysmorphic sacral morphology and cortical breach risk, the present study was not powered to define validated safety thresholds for these parameters. Therefore, these measurements should be interpreted as risk-associated morphometric indicators rather than definitive cutoff values. Larger multicenter studies with ROC-based validation are needed to establish clinically reliable threshold values for screw anteversion and corridor width.

Sex-related differences in pelvic anatomy may further influence fixation safety. In the present study, female patients demonstrated narrower S1 corridor dimensions compared with males across both morphology groups, whereas anteversion angles were largely comparable. These findings are consistent with prior morphometric studies reporting less favorable osseous anatomy for iliosacral fixation in females and a higher prevalence of dysmorphic features [24,25,26]. Although Balling et al. suggested that transsacral feasibility may be largely independent of sex [27], corridor width may represent an additional anatomical constraint in female patients. Given the limited sample size of subgroup analyses, these findings should be interpreted with caution.

The present findings indicate that sacral dysmorphism is not merely an anatomical variation but a clinically significant factor associated with an increased risk of cortical breach during S1 iliosacral screw fixation. These results highlight the importance of careful CT-based assessment of sacral morphology and emphasize the need for individualized trajectory planning in patients with dysmorphic anatomy. Preoperative identification of corridor narrowing and increased anteversion requirements may assist surgeons in selecting the most appropriate fixation strategy and in anticipating technical challenges during screw placement.

The present study has several strengths, including the use of routine high-resolution CT imaging, which enabled detailed morphologic assessment and standardized corridor measurements. The use of routine postoperative CT imaging in all patients further represents a methodological strength, as it enabled unbiased detection of cortical breach independent of clinical presentation. In addition, all procedures were performed by a dedicated trauma team, minimizing variability related to surgical technique and reducing the influence of the learning curve.

Nevertheless, several limitations should be acknowledged. The retrospective single-center design may limit generalizability and introduces the potential for selection bias. Furthermore, intraoperative fluoroscopic visualization could not be fully standardized, as factors such as soft-tissue conditions and bowel gas may affect image quality. Finally, subgroup analyses, particularly those based on sex and sacral morphology, were limited by sample size and should be interpreted with caution. Although screw diameter and selection technique were standardized, minor intraoperative adjustments in screw length or trajectory based on individual anatomy may have occurred and cannot be entirely excluded.

Future research should focus on prospective validation of these findings in larger, multicenter cohorts with standardized imaging protocols. In particular, studies incorporating receiver operating characteristic (ROC) analysis are needed to establish clinically meaningful threshold values for screw anteversion and S1 corridor width. In addition, the integration of advanced imaging techniques and navigation-assisted fixation strategies may further improve the accuracy and safety of iliosacral screw placement in dysmorphic sacra.

From a practical standpoint, the routine incorporation of CT-based sacral morphology assessment into preoperative planning is essential for identifying high-risk anatomical patterns. This approach may facilitate more precise trajectory optimization, reduce the likelihood of cortical breach, and ultimately improve the safety and reliability of iliosacral screw fixation in patients with dysmorphic sacra.

Taken together, these findings support the concept that sacral dysmorphism is not merely an anatomical variation but a clinically actionable factor that should directly influence surgical strategy.

## 5. Conclusions

Sacral dysmorphism is an independent risk factor for cortical breach during percutaneous S1 iliosacral screw fixation. The geometric constraints of dysmorphic sacra, including corridor narrowing and increased anteversion requirements, significantly compromise screw placement safety. Careful CT-based evaluation and individualized trajectory planning are therefore essential to optimize fixation outcomes in this high-risk anatomical subgroup.

## Figures and Tables

**Table 1:** Values are presented as medians with interquartile ranges (IQR) and means with standard deviations (SD), as appropriate.

Sacral Morphology	Sex	*n*	Screw Anteversion (°)	Narrow Corridor Width (mm)
			Median (IQR) [Mean ± SD]	Median (IQR) [Mean ± SD]
Normal	Female	25	8 (5–11) [8.0 ± 4.49]	18 (14–22) [18.0 ± 4.95]
	Male	55	9 (8–9) [8.89 ± 2.23]	26 (24.5–27.5) [25.98 ± 4.99]
Dysmorphic	Female	14	28.5 (24.5–32.5) [28.5 ± 4.74]	14 (10.5–17.5) [14.0 ± 3.33]
	Male	18	26.5 (22.25–30.75) [26.67 ± 4.95]	17 (13.25–20.75) [17.0 ± 4.23]

Note: Values are presented as medians with interquartile ranges and means with standard deviations. Statistical comparisons were performed using the Mann–Whitney U test.

## Data Availability

The data supporting the findings of this study are available from the corresponding author upon reasonable request.
